# The burden of hospital-acquired legionellosis in German teaching hospitals

**DOI:** 10.1007/s15010-026-02782-2

**Published:** 2026-04-01

**Authors:** Stefanie Kramme, Winfried Ebner, Anne Lösslein, Barbara Maier, Christian Schneider, Jan Liese, Christian Brandt, Alexandra Heininger, Heike von Baum, Tjibbe Donker, Sandra Reuter, Philipp Henneke

**Affiliations:** 1 Present Address: Institute for Infection Prevention and Control, Medical Center, Faculty of Medicine, University of Freiburg, Breisacher Str. 115B, 79014 Freiburg, Germany; 2 Institute for Medical Microbiology and Hygiene, Medical Center, Faculty of Medicine, University of Freiburg, Freiburg, Germany; 3 Institute of Medical Microbiology and Hygiene, Tübingen University Hospital, Tübingen, Germany; 4 Institute of Medical Microbiology and Hygiene, Ulm University Hospital, Ulm, Germany; 5 Department of Hygiene, Medical Faculty Mannheim, Mannheim University Hospital, University of Heidelberg, Mannheim, Germany; 6 Dept. of Infectious Diseases, Medical Faculty Heidelberg, Medical Microbiology and Hygiene, Heidelberg University, Heidelberg, Germany; 7 Centre for Planetary Health, University of Freiburg, Freiburg, Germany; 8 Centre for Chronic Immunodeficiency, Medical Center, Faculty of Medicine, University of Freiburg, Freiburg, Germany

**Keywords:** *Legionella pneumophila*, Hospital-acquired, Nosocomial, Case report, Survey, Infection prevention

## Abstract

**Purpose:**

*Legionella pneumophila* is a recognized cause of hospital-acquired pneumonia particularly in immunocompromised patients. Significant resources are dedicated to the monitoring and control of Legionella contamination in hospital water systems. However, the actual risk of nosocomial Legionella infections in German university hospitals, which represent large tertiary care institutions, remains poorly defined. The aim of this study was to quantify the incidence of nosocomial legionellosis in German university hospitals. In addition, the extent of routine testing of hospital drinking water systems and preventive measures implemented on legal grounds was investigated.

**Methods:**

We conducted a retrospective analysis of nosocomial Legionella cases over a five-year period (2020—2024) at the University Medical Center Freiburg (UMCFR), including in-depth genomic typing. We distributed a survey to all 36 German university hospitals to collect data on the incidence of nosocomial infections and prevention strategies. We collected comprehensive data on preventive measures on a regional level among university hospitals in Baden-Württemberg.

**Results:**

Over a five-year period at UMCFR, three patients met the criteria for nosocomial Legionnaires’ disease (LD). In two of these cases, we were able to confirm transmission through the hospital’s drinking water system by genome analysis. Nationwide, 26 of 36 University hospitals reported a total of sixteen cases. All hospitals documented abnormal parametric values (≥ 100 CFU/ml) in their drinking water systems at least once per year. Preventive measures varied widely: the number of sampling points ranged from 30 to 2355, and the number of point-of-use water filters from 30 to 2,200 per hospital. Despite this, incidence of LD remained very low.

**Conclusions:**

Nosocomial Legionella infections are rare in German tertiary care hospitals. However, the impact of intensive prevention measures on the incidence rates of nosocomial Legionella infections remains unclear. Therefore, current practices should be re-evaluated to optimize the use of resources without compromising patient safety.

**Supplementary Information:**

The online version contains supplementary material available at 10.1007/s15010-026-02782-2.

## Introduction

Legionella spp. naturally occur in freshwater environments. They have co-evolved with protozoa, which facilitates their adaption to eukaryotic cells [1, 2]. *Legionella pneumophila* (*L. pneumophila*) was first identified as a human pathogen in connection with a severe pneumonia outbreak at the 1976 American Legion convention in Philadelphia, which led to the denomination Legionnaires' disease [3]. In Germany, approximately 10–15% of reported Legionnaires’ disease cases are nosocomial [4, 5]. Across Europe, ELDSNet data indicate that 9.6% of locally acquired cases between 2008 and 2017 were healthcare-associated [4, 5]. Hospital-acquired *Legionella pneumophila* infections are typically traced to colonized hot-water systems. Transmission occurs mainly through inhalation of aerosols generated from contaminated water sources such as showers, taps, dead-end pipes, fountains, or hydrotherapy equipment [2, 6–8]. Recent evidence suggests that aerosols produced during showering represent a significant exposure pathway. In an outbreak investigation conducted at a university hospital between 2018 and 2021, showering during hospitalization was identified as an independent risk factor for hospital-acquired Legionella pneumonia, classifying it as a modifiable risk [7, 9, 10].

As indicated already at the time of discovery, *L. pneumophila* is a particular threat to immunocompromised individuals, i.e. the elderly and, possibly even more so to patients with primary and secondary immunosuppression. The intracellular lifestyle of the pathogen with a preference for innate immune cells, i.e. macrophages, interferes with the elimination of the pathogen [11]. The burden of *L. pneumophila* pneumonia in the general population is considered substantial with an estimated 15,000–30,000 cases per year in Germany [12]. In contrast, the incidence of nosocomial infections, i.e. acquired in hospitals by high-risk patients, remains relatively low. However, these infections pose a major threat to health as they are associated with a mortality rate that is almost three times higher than that of community-acquired infections, reaching up to 30% [13]. In recent years, the incidence of Legionnaires’ disease has been increasing in North America and Europe [14, 15]. Risk factors for severe disease include male gender, age over 50 years, history of smoking, alcohol abuse, hematological malignancies, and immunosuppression. In Germany, Legionnaires’ disease is a notifiable condition, yet the cases reported to the health authorities most likely underestimate the disease incidence by far [16]. At the European level the age-standardised notification rate of Legionnaires’ disease was 1.8 to 2.2/100,000 from 2017 to 2019, with a remarkable 30% increase as compared to the first half of the last decade [17].

In view of these risks, there are strict regulatory frameworks in place, such as those of the German “Ordinance on the Quality of Water Intended for Human Consumption”, which requires regular tests for Legionella in drinking water systems with at least annual testing mandated in hospitals. Similar testing obligations are applying to other types of buildings covered by the ordinance but with sampling intervals up to three years if no Legionella contamination could be detected.

Even though the detection of Legionella spp. does not necessarily indicate an immediate increase in transmission risk, German regulations require that exceedance of the technical action value of 100 CFU/100 ml triggers a structured, risk-based assessment and the implementation of appropriate control measures according to established technical standards.These include filtration of water outlets, flushing protocols, repeated sampling, and disinfection of the water system by heat or chemicals. Such measures demand substantial human and financial resources.

In view of these uncertainties, we undertook three complementary approaches to address gaps in knowledge about nosocomial Legionella infections. First, the occurrence of two nosocomial Legionella infections in 2022 and 2023, which met the case definition and could be confirmed by molecular typing, prompted a retrospective analysis of all Legionella-positive laboratory results from 2020 to 2024, during which no additional nosocomial cases were identified.Second, we designed an ad-hoc survey of all university hospitals in Germany to characterize the epidemiology and existing measures in place to prevent healthcare-associated Legionella infections. Finally, we used the Infection Control Network (BW-HIP) of all university hospitals in the federal state of Baden-Württemberg, which has a population of over 11 million. The aim was to explore the relationship between the incidence of nosocomial Legionella infections and the implementation of preventive measures.

## Methods

### Analysis of nosocomial Legionella cases at the University Clinic Freiburg

#### Case definition

A case was defined by a) identification of *L. pneumophila* in respiratory tract specimens (culture or PCR) or urine samples (antigen test) or antibody detection by immunofluorescens test for *L. pneumophila* serogroup 1 *plus* b) clinically confirmed pneumonia or disease-related death. Cases were classified as healthcare-associated if patients met established criteria (RKI, 2025) and the patients remained in a medical facility during the period of probable infection, which corresponds to the incubation period of 2–10 days.

#### Extraction of Legionella cases from patient management system

Positive test results were automatically and routinely reported to the infection control department by the Institute for Medical Microbiology. Subsequently, an epidemiological investigation is conducted, including consultations with the relevant department to address clinical details, particularly if nosocomial Legionellosis must be suspected.

#### Detection and Sampling of Drinking Water Sources in Suspected Nosocomial Legionella Infections

If the clinical, diagnostic, and epidemiological criteria suggested a nosocomial Legionella infection, the patient management system was used to identify the rooms where the patient was located. In the relevant areas, water samples were collected from the existing outlets. These samples were then analyzed for Legionella spp. In the hospital hygiene laboratory using the methods described below.

#### Laboratory investigations

Culture-based identification of *L. pneumophila* was performed by standardized protocols at the local clinical microbiology laboratories. In Freiburg, this involved inoculation on GVPC agar plates comprising buffered charcoal yeast extract with L-cysteine, glycine, vancomycin, polymyxin B, and cycloheximide (Thermofisher, Wesel, Germany) and culture for 10d at 36 °C, with 5% CO_2_ (vol/vol) atmosphere for the first 48 h and in moist room air atmosphere thereafter. Identification of cultured isolates was performed using MALDI-TOF mass spectrometry (Bruker Daltonics GmbH & Co. KG, Bremen, Germany).

For molecular detection of Legionella in respiratory samples, DNA was extracted using QIAamp DNA Mini Kit (Qiagen, Hilden, Germany) and tested in the real time PCR panel Allplex™PneumoBacter Assay (Seegene, Düsseldorf, Germany). Urinary antigen detection was conducted using a commercially available Legionella Fluorescent Immunoassay (Sofia^®^ Legionella FIA, Quidel, Köln, Germany) using immunofluorescence-based lateral-flow technology and a fluorescent immunoassay analyzer according to the manufacturer’s instructions (Sofia^®^, Quidel, Köln, Germany)). Positive results were confirmed by repeated testing after heating urine specimen at 95 °C for 5 min followed by a 15-min centrifugation step (1000 X g) to avoid false positive results due to the presence of rheumatoid-like factors in the samples.

All isolates from positive cases were confirmed by the German reference laboratory in Dresden, Germany.

### Whole genome sequencing (WGS)

DNA extraction from cultured isolates was carried out using the Roche High Pure Template Preparation kit. Library preparation was conducted using the Nextera DNA Flex Library Prep according to the manufacturer’s protocols. Sequencing was performed with 2 × 150 bp paired-end sequencing on an Illumina MiSeq, and raw data were processed using established bioinformatics pipelines for quality control, assembly, and phylogenetic analysis. Raw sequence reads were mapped to the reference genome Philadelphia (ATCC 33152, accession AE017354) using smalt [18]. Subsequent variant filtering was carried out using samtools [19] in combination with the Genome Analysis Toolkit [20]. The minimum accepted coverage was 30X per sample. De novo assemblies were generated using SPAdes v3.13.1 [21] with kmer sizes 21, 33, 55, 77, 99, 109, and 123, followed by filtering to only include contigs with a minimum of 500 bp. Assemblies were of accepted quality if they were of expected size (3-4 Mb), number of contigs less than 500, largest contig greater than 100,000 bp, and N50 greater than 100,000 bp. Kraken with mini-kraken database [22] was used to check the species and potential contamination. Sequence-based typing was performed using legsta (https://github.com/tseemann/legsta). The average coverage was 72X, with a genome size of 3.3 Mb, N50 of 466,500 bp, and median number of 46 contigs. For phylogenetic inference, an alignment file was generated from mapping to an in-cluster reference. Recombination was removed using gubbins [23], visualized in Artemis [24] and resulting trees were visualized using figtree v1.4.4 (https://github.com/rambaut/figtree). Detailed metadata can be found in supplementary table S1 and sequencing data has been depositied in ENA project PRJEB104143 with individual accession numbers in table S1.

### Survey of university hospitals

A structured questionnaire was developed to assess the epidemiology of nosocomial Legionella cases and implemented control measures across German university hospitals. We distributed the questionnaire via the Association of German University Hospitals (Verband der Universitätsklinika Deutschlands https://www.uniklinika.de/) via email to infection control departments of all 36 university medical centers in spring 2024. Recipients were requested to return completed surveys within four weeks. The questionnaire covered topics including incidence of cases, frequency of drinking water testing, water system interventions, and usage of point-of-use filters. The full questionnaire is provided in the supplementary material (Supplementary file 1).

### Survey of Baden-Württemberg University Hospitals

To complement the survey, additional details were collected from the Baden-Württemberg university hospitals located in the state of Baden-Württemberg: the number of drinking water samples for Legionella testing taken annually from 2020 through 2024, the number of abnormal parametric values recorded based on these samples and the number of point-of-use filters installed between 2020 and 2024.

## Results

An analysis of the electronic patient data management system of the Medical Center—University of Freiburg covering the years 2020—2024 identified three recent cases of hospital-acquired *L. pneumophila* pneumonia in our university hospital in 2022 and 2023, respectively.

### Case 1

A 61-year-old male patient with a history of aortic aneurysm was admitted for implantation of an aortic Y-prosthesis at University Medical Centre Freiburg. Underlying health risk factors included hypertension, nicotine abuse, and obesity. The surgery proceeded without complications, and the patient was transferred to the cardiac and vascular surgical intensive care unit for further monitoring and treatment. The patient was extubated without complications. the patient was transferred to the general ward, exhibiting good gas exchange, sufficient spontaneous breathing, and intact protective reflexes. During the subsequent days, the patient developed increasing respiratory insufficiency of mixed etiology (clinically suspected pneumonia in addition to hypervolemia and atelectasis) and was readmitted to the intensive care unit. At that time empiric antibiotic therapy was initiated with piperacillin/tazobactam, subsequently switched to Clarithromycin and Levofloxacin. Due to further clinical deterioration, the patient required intubation and invasive mechanical ventilation starting followed by ECMO (Extracorporeal membrane oxygenation).

Due to the respiratory deterioration a bronchoalveolar lavage (BAL) specimen obtained on postoperative day 13 showed growth of *L. pneumophila* on postoperative day 20 whereas a urinary antigen test conducted on day 15 post-op yielded a negative result. In-house whole genome sequencing (WGS) identified *L. pneumophila* sequence type (ST) 93 which was additionally confirmed by the national reference center in Dresden together with the additional information that *L. pneumophila* serogroup 3 was proved. On day 24 post-op the patient died from septic shock, most likely due to a combination of nosocomial pneumonia and *Escherichia coli* septicaemia. WGS analysis of a cold sink tap water sample from the patient’s room on the peripheral ward revealed *L. pneumophila* serogroup 3, ST93, so that the hospital cold water system must be regarded as the probable source of infection. The cold water had to be sampled by order of the public health department after the cold water temperature had previously been exceeded. The shower drain was equipped with a bacterial filter at the time of potential pathogen transmission. The patient stayed for 4 days in the patient room on the general ward, in which Legionella had been detected, prior to the planned surgical intervention and developed documented respiratory deterioration 5 days after leaving this room, which made admission to the intensive care unit necessary.

### Case 2

A 74-year-old female patient with a history of vaginal mucosal melanoma, autoimmune thyroiditis, hepatitis, and colitis was admitted to the dermatology clinic following radiotherapy. She was discharged 15 days later under treatment with nivolumab, ipilimumab, and methylprednisolone. Subsequently, she contracted COVID-19 and experienced a clinical deterioration 4 days after discharge from the hospital and was readmitted. After another three days the urinary antigen test specific for *L. pneumophila* serogroup 1 and PCR detecting *L. pneumophila* DNA were positive. The sputum culture taken on day 4 after readmission yielded *L. pneumophila.* In the In-house WGS of isolates from tap water and shower samples from the patient’s room *L. pneumophila* ST93, was detected identical to that found in the patient´s respiratory specimen. This result was confirmed by the national reference center in Dresden, where serogroup 3 was also identified. These findings strongly suggest that the hospital drinking water system was the probable source of infection. The patient received a six-day course of levofloxacin started on day 3 post-admission followed by seven days of piperacillin/tazobactam due to suspected bacterial superinfection, resulting in clinical improvement and discharge.

**Fig. 1:**
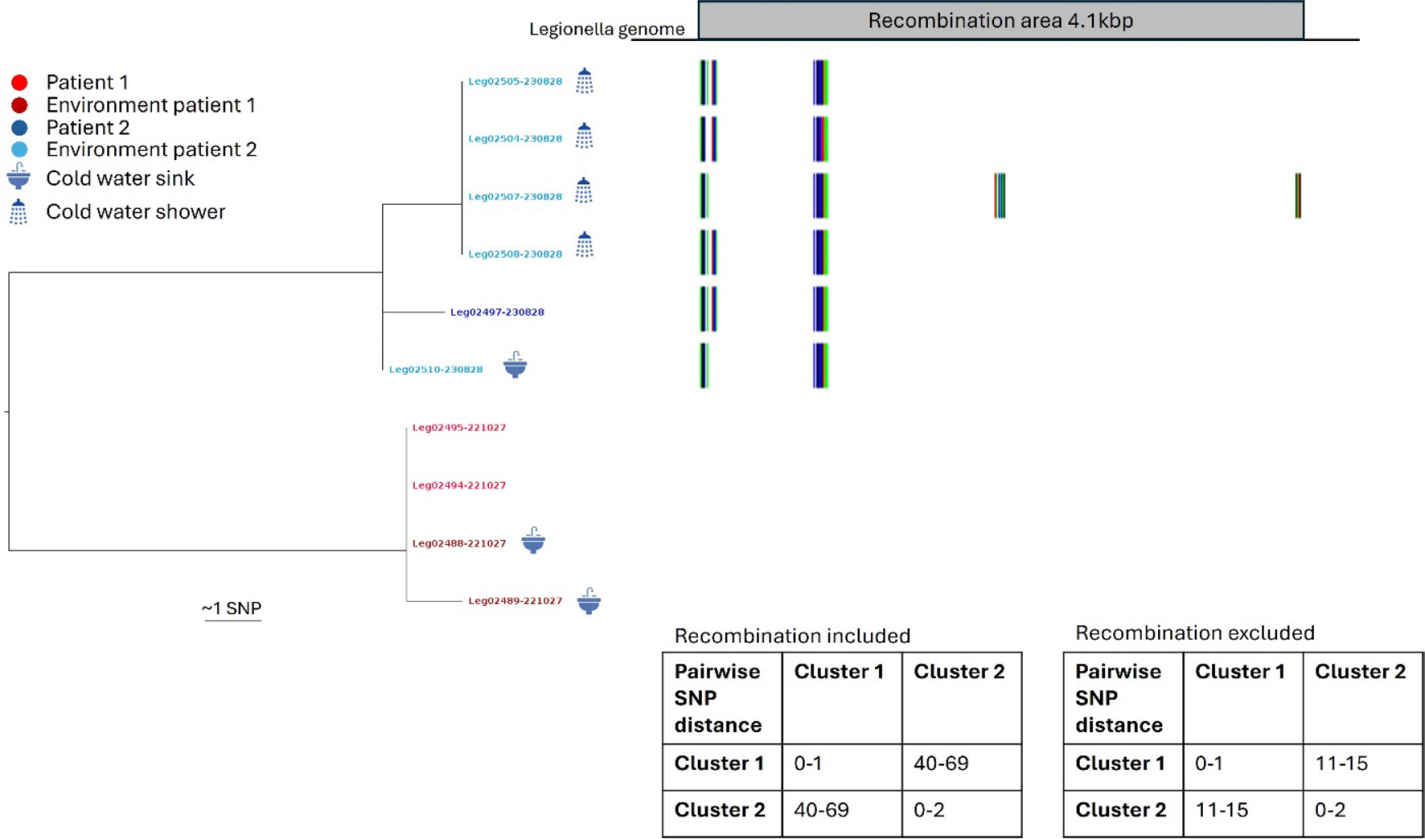
Phylogenetic reconstruction of ST 93 isolates and visualization of recombination. The phylogeny is based on mapping to within-cluster reference Leg02494, and the tree is mid-point rooted. Patient 1 and their environment are coloured red, patient 2 and their environment are coloured blue. Water sources are indicated by symbol and colour. The right panel shows the region encompassing the recombination are, with SNPs indicated by different colours corresponding to the bases (A green, C red, G blue, T black). The two tables show pairwise distance within and between clusters including and excluding recombination

Since both cases and the corresponding water samples implicated serogroup 3-ST93, the available isolates were compared (Fig. 1), ignoring the fact that the two patients were housed in buildings that are geographically distant from each other and supplied by entirely independent drinking-water systems. We found that within the patient-environment samples, there were 0–2 SNPs, indicating identical isolates, however, between the two events we found 11–15 SNPs. A recombination event was observed in the later cluster of isolates. Previous studies have found 14 SNPs in isolates from France 2011 and Australia 2013 [25], hence we conclude these two cases to be unrelated genomic-epidemiologically.

As a consequence of the aforementioned nosocomial Legionella cases, authorities demanded far-reaching measures. These included a risk assessment by an external expert, increasing the number of water outlet samples (including cold-water samples) to be analyzed for Legionella contamination, installing bacterial filters on all affected wards, and implementing decontamination measures. Moreover, a Water Safety Team was formed, consisting of experts from sanitary engineering, occupational safety, and hospital hygiene, to regularly discuss all tap-water–relevant issues and to initiate and control appropriate measures.

### Results of the national Ad Hoc Questionnaire

**Fig. 2:**
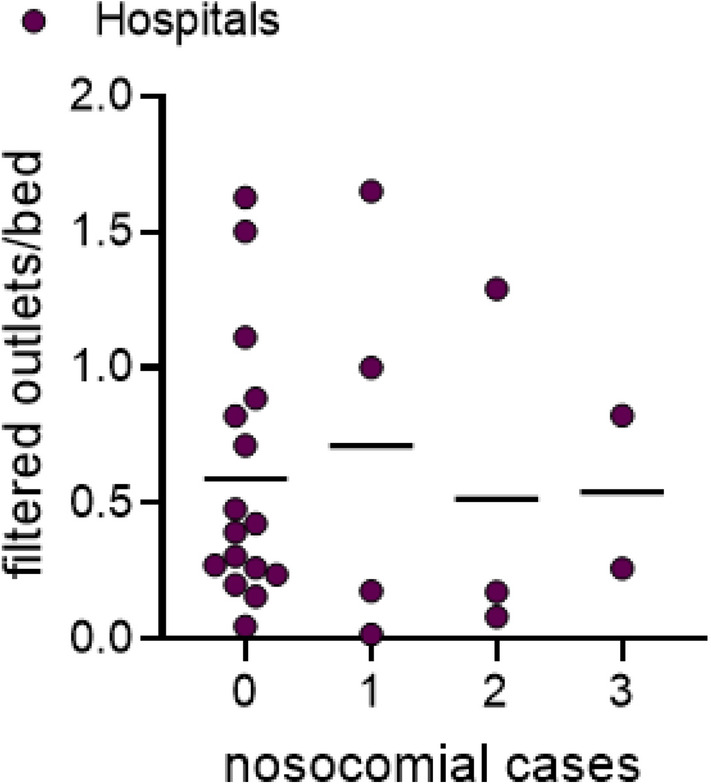
Number of nosocomial Legionella cases and filtered water outlets per hospital bed capacity. No association could be demonstrated between the number of filters per water outlet and the occurrence of nosocomial Legionella infections. Hospitals with nosocomial cases reported both low and high filter counts, and vice versa

To gain an epidemiological overview, an ad hoc questionnaire was distributed to all 36 university hospitals in Germany. Overall, 26 out of 36 hospitals provided data, which represents a response rate of 72%. Over five years (2020–2024), nine hospitals reported a total of 16 nosocomial Legionella infections, corresponding to an average incidence of approximately 0.123 cases per hospital per year or 1 infection per hospital per 8.1 years (0,26 cases/100000 patients). Accordingly, hospital-acquired legionellosis is a rare, sporadic infection entity in Germany. Notably, all hospitals reported at least one abnormal parametric value for Legionella spp. (100 cfu/100 mL) per year. This showed that the pathogen widely persists in the hospital drinking water systems despite the measures required by legal standards after exceeding the parametric values. The German university hospitals also reported on the implementation of measures, including flushing, closure of water outlets and repairs to the water supply. All German university hospitals applied water outlet filters at least in high-risk units such as oncology wards, transplant centers, adult and pediatric intensive care units, dialysis units, and delivery rooms with the number of filtered outlets ranging from 30 to 2,200 per hospital. We did not find an association of nosocomial Legionella infections and number of bacterial filters (Fig. 2). The frequency of water sampling varied considerably, with the number of annual samples per hospital ranging from 114 to 2,355.

## Results of the regional Survey in Baden-Württemberg

A focused survey of the five university hospitals in Baden-Württemberg identified a total of six nosocomial Legionella infections within the five-year period, all of which occurred in two hospitals. With an estimated total of approximately 1.5 million inpatient admissions in these hospitals over the same period, this corresponded to an incidence of around 0.4 cases per 100,000 admissions (≈1 case per 250,000 patients).

**Fig. 3:**
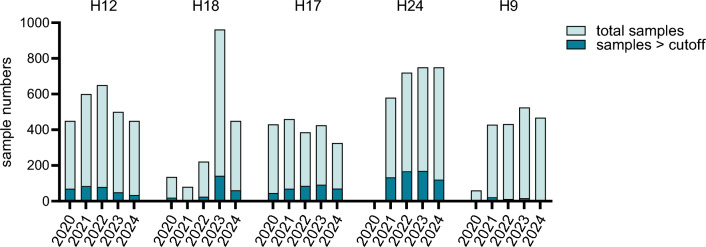
Total number of water samples taken and parametric values > cutoff per year at five university hospitals in Baden-Württemberg, Germany

**Fig. 4:**
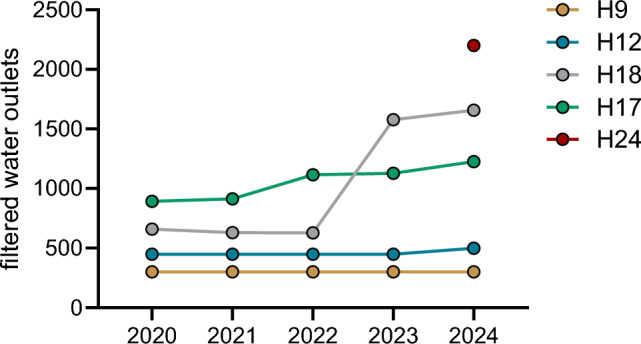
Number of documented filtered water outlets per year in five university hospitals in Baden-Württemberg, Germany

No significant correlation was found between the number of water outlet filters and the incidence of nosocomial cases, nor between the number of water samples taken and the proportion of exceedances amonst these samples (Figs. 3, 4), underscoring the complex, multifactorial nature of Legionella transmission and the limitations of filtration alone as a preventive measure.

## Discussion

Nosocomial Legionella infections remain a concern due to higher mortality compared with community-acquired infections. Although studies from Europe and Germany have reported proportions ranging from 5.1% to 13.6% for nosocomial legionellosis between 2008 and 2017, our investigation, which focused exclusively on university hospitals in Germany, revealed a considerably lower incidence. Across six university hospitals, a total of 16 nosocomial legionellosis cases were identified over a five-year period. The accurate detection of nosocomial Legionella infections is likely impeded by the non-specific clinical presentation of affected patients, which often leads to the early administration of empirical antibiotic therapy. This, in turn, compromises laboratory diagnostics that ideally require the cultivation of these fastidious bacteria in addition to the Legionella antigen test and PCR from lower respiratory specimens. Consequently, a substantial underreporting of both community-acquired and nosocomial legionellosis must be assumed in general, and an underestimation of the true burden cannot be excluded for this survey either [26].

Given the increased virulence of mAb 3/1–positive *L. pneumophila* strains, there is a need for more widely available diagnostics to effectively address both clinical cases and the potential higher risks within the drinking-water network.

For both cases acquired at our hospital in the analysis time period and explained in detail above, diagnosis of nosocomial legionellosis was established using a range of laboratory techniques. We found that high-resolution diagnostic approaches, including whole-genome sequencing (WGS), can be instrumental in clarifying transmission pathways. Comparative analysis of WGS data from cultured Legionella isolates obtained from clinical specimens and from hospital water sources enables definitive confirmation of nosocomial transmission. Furthermore, WGS data analysis demands substantial technical expertise and bioinformatics infrastructure, which may be inaccessible to many laboratories [27]. Even in well-equipped facilities, the diagnostic yield can be compromised by prior antibiotic administration or suboptimal sample quality. [26].

Our survey shows that exceedances of the technical action value for *Legionella* in drinking water are a regular and expected occurrence, found in 10–23% of the water samples examined in university hospitals in Baden-Württemberg. For Germany, Dilger et al. [4] reported a prevalence of 20.7%, and Kruse et al. [5] detected *Legionella* spp. in 32.7% of the water samples collected from 712 buildings (not limited to hospitals), which supports the findings of our survey. Regarding species identification, which is important for assessing pathogenicity, Dilger et al. found *L. pneumophila* in 83.85% of positive samples.

The interpretation of Legionella parametric value in drinking water systems remains difficult, as a direct correlation between the number of detected Legionella colony-forming units and the risk of pathogen transmission or even symptomatic disease lacks a robust evidence base. In contrast, authorities often impose rigid regulatory measures when an abnormal parametric value is reported. Legionella spp. densities in water samples show substantial temporal fluctuations [28]. Furthermore, it could be demonstrated that different genotypes of Legionella spp. with distinct virulence markers and specific temperature-dependent growth behaviors could be detected at various sites in the same drinking water network. A detailed assessment of Legionella diversity within a drinking water system may enable a more differentiated interpretation of drinking water findings and their implications [29].

Given the optimal growth temperature range of Legionella between 25 °C and 50 °C, maintaining appropriate water temperatures within hospital plumbing systems is a critical prevention measure. The risk of Legionella contamination increases with the age, the complexity, and size of the water system. Factors such as limited water flow, e.g. in times of water scarcity, and routine maintenance can disrupt temperature control at peripheral outlets, thereby promoting bacterial growth. Next to favorable temperatures, biofilm formation, and the presence of eukaryotic hosts such as protozoa that facilitate bacterial survival and replication are key drivers of Legionella growth in drinking water [30]. Importantly, biofilms represent a significant barrier to eradication by thermal and chemical disinfection measures. Additionally, water stagnation within complex plumbing networks, which are common in healthcare settings, exacerbates these problems thus complicating infection control efforts.

To specifically highlight and broadly quantify one preventive measure namely the implementation of point-of-use filters we selected the use of point-of-use (POU) end-of-line water filters as a quantifiable intervention parameter. Our nationwide survey showed that all German university hospitals use POU filters both in high-risk areas (e.g. oncology, transplantation) and as an acute measure in response to exceedances of the parametric value. To our knowledge, no comparative quantitative data on POU filter use in Germany or Europe are available. A longitudinal analysis of university hospitals in Baden-Württemberg indicates that, as expected, POU filter use increases in association with nosocomial infections, in addition to other measures (e.g. risk-based intensified sampling, pipeline modifications).

In addition, the genomics of Legionella spp. is a challenge as their evolution occurs mainly by recombination [31]. This means that epidemiologically, temporally and geographically unrelated isolates may differ only by a few SNPs compared to Enterobacterales species such as *Escherichia coli* and *Klebsiella pneumoniae*. As an example, isolates from different continents and years (France 2011 and Australia 2013) differed only by 14 SNPs in one study [25]. In contrast, such a small genetic distance in other species, e.g. *K. pneumoniae*, would most likely indicate that they are isolates from the same hospital [32]. In the cases described here, including recombination led to 40–70 SNPs distance between the clusters, removing recombination reduced this to 11–15 SNPs. These genetic peculiarities must be taken into account in the evaluation of Legionella outbreaks. Overall, we found a statistically hard-to-define risk of nosocomial Legionella infection even in tertiary care centers serving severely ill and immunocompromised patients. Although extensive implementation of outlet filtration can effectively reduce the risk of patient contamination from drinking water, it may also result in reduced flow rates in drinking water pipes and, consequently, deterioration of drinking water hygiene, including increased biofilm formation. Moreover, the filters represent a considerable financial burden.

These considerations raise important questions about cost-effectiveness and resource allocation for Legionella surveillance and control, underscoring the need for risk-adapted strategies that balance rigorous prevention with practical feasibility.

The limitations of this study include the small number of nosocomial legionellosis cases observed at the university hospitals, which may already reflect the effectiveness of implemented preventive measures, such as filtration in high-risk areas. Furthermore, only university hospitals were included, where, on the one hand, a higher number of at-risk patients would be expected, but on the other hand, diagnostic procedures should be readily accessible and up-to-date through affiliated microbiology laboratories. However, this is not reflected in the observed incidence rates. In addition, the data were not collected prospectively, which may have contributed to the lower number of cases identified through the retrospective study design.

## Conclusion

Healthcare-associated infections caused by Legionella spp. are particularly concerning due to high mortality rates, which significantly exceed those of community-acquired cases. These infections may serve as indicators of infrastructural and management problems within hospital settings.

However, despite the frequent detection of Legionella spp. in drinking water systems, such infections remain exceedingly rare in large German university hospitals that care for the most susceptible patient populations. Infection prevention strategies vary considerably between hospitals, yet no clear impact on infection incidence has been observed. The low number of cases, however, limits the ability to draw definitive conclusions. Overall, there is a need for a throrough evaluation of the impact of existing intensive prevention measures on the low incidence of hospital-acquired Legionella cases.

## Supplementary Information

Below is the link to the electronic supplementary material.

Supplementary file1 (DOCX 17 KB)

## Data Availability

Sequencing data has been deposited in ENA project PRJEB104143 with individual accession numbers in table S1.
