# Supplementary material for: The burden of hospital-acquired legionellosis in German teaching hospitals
Source: Infection. 2026 Apr 1;54(3):1455–63. doi: 10.1007/s15010-026-02782-2 (PMC13323518; doi:10.1007/s15010-026-02782-2)
Supplement: Supplementary file 1 — Supplementary file1 (DOCX 17 KB) [file 15010_2026_2782_MOESM1_ESM.docx]

**Supplement**

|  | **2020** | **2021** | **2022** | **2023** | **2024** |
| --- | --- | --- | --- | --- | --- |
| **Hospital 12 Water sampling** | 450 | 600 | 650 | 500 | 450 |
| **12 abnormal parametric values** | 70 | 85 | 80 | 50 | 35 |
| **Hospital 18 Water sampling** | 136 | 80 | 222 | 962 | 450 |
| **18 Parametric values** | 20 | 3 | 25 | 143 | 61 |
| **Hospital 17 Water sampling** | 430 | 460 | 386 | 425 | 326 |
| **17 abnormal parametric values** | 46 | 70 | 86 | 93 | 71 |
| **Hospital 24 Water sampling** | 0 | 580 | 720 | 750 | 750 |
| **24 abnormal parametric values** | 0 | 134 | 168 | 170 | 121 |
| **Hospital 9 Water sampling** | 60 | 429 | 432 | 525 | 468 |
| **9 abnormal parametric values** | 1 | 23 | 13 | 17 | 8 |

**Table 1** (corresponding to figure 3)**:** Total number of water samples taken and abnormal parametric values per year at four university hospitals in Baden-Württemberg, Germany.

|  | **2020** | **2021** | **2022** | **2023** | **2024** |
| --- | --- | --- | --- | --- | --- |
| **Hospital 12** | 450 | 450 | 450 | 450 | 500 |
| **Hospital 18** | 660 | 630 | 629 | 1578 | 1657 |
| **Hospital 17** | 893 | 913 | 1115 | 1128 | 1227 |
| **Hospital 24** | 0 | 0 | 0 | 0 | 2200 |
| **Hospital 9** | 300 | 300 | 300 | 300 | 300 |

**Table 2** (corresponding to figure 4)**:** Number of documented filtered water outlets per year in five university hospitals in Baden-Württemberg, Germany.
